# The rollercoaster of obsessive-compulsive disorder: How chronotype and time of day affect behavioral inhibition in adults with OCD^☆^

**DOI:** 10.1016/j.xjmad.2025.100113

**Published:** 2025-03-01

**Authors:** Eyal Kalanthroff, Yuval Seror, Noa Sagi, Shachar Hochman, Omer Linkovski, Hadar Naftalovich, Helen Blair Simpson

**Affiliations:** a Department of Psychology, The Hebrew University of Jerusalem, Israel; b Department of Psychiatry, Columbia University, New York, NY, United States; c School of Psychology, University of Surrey, Guildford, UK; d Department of Psychology & Gonda Multidisciplinary Brain Research Center, Bar-Ilan University, Ramat-Gan, Israel; e New York State Psychiatric Institute, New York, NY, United States

**Keywords:** Obsessive-compulsive disorder, Inhibitory control, Chronotype, Alertness, Symptom-provocation

## Abstract

Chronotype, a person's circadian preference, reflects individuals’ natural pattern of alertness levels throughout the day. It has been shown that chronotype contributes to within-day symptom fluctuations in obsessive-compulsive disorder (OCD). However, the underlying mechanism of this effect is not yet clear. Inhibitory control, an executive function crucial for suppressing unwanted behaviors and thoughts, is essential for managing OCD symptoms and is highly influenced by alertness levels. Hence, the current study investigated the impact of chronotype and time of day on inhibitory control in OCD patients, particularly in response to individually tailored symptom-provoking images, using a novel version of the stop signal task. Ninety-three treatment-seeking OCD patients completed an individually tailored Symptom-Provocation Stop-Signal Task, every morning and evening for four consecutive days. Chronotype was assessed using the Morningness-Eveningness Questionnaire (MEQ). Stop-signal reaction time (SSRT) was assessed for neutral and individually tailored symptom-provoking images separately. Patients exhibited longer SSRTs (worse inhibition) to symptom-provoking trials compared to neutral trials. Most importantly, chronotype and time of day significantly interacted to affect SSRTs in the symptom-provocation condition: A significant correlation was found between optimal alertness periods and improved inhibitory control in the symptom-provocation condition. Taken together, these results indicate that inhibitory control in OCD varies with chronotype and time of day. These findings suggest that aligning treatment sessions with patients' chronotype may enhance therapeutic outcomes and that heightening alertness—by increasing the chances of successful inhibition—might be a way to mitigate the vicious cycle of OCD.

## Highlights

• We investigated why chronotype and time of day influence symptom fluctuation in OCD.
• Patients completed a tailored Symptom-Provocation Stop-Signal Task for four days.
• OCD patients show worse inhibition to symptom-provoking vs. neutral stimuli.
• Optimal alertness improves inhibition during symptom-provocation in OCD patients.
• Heightened alertness could help break the vicious cycle of OCD compulsions.

Obsessive-compulsive disorder (OCD) affects approximately 1 in 40 individuals. It is characterized by distressing-repetitive thoughts (obsessions) and repetitive actions or mental acts (compulsions) to alleviate distress [1]. While compulsions may provide momentary relief, they perpetuate a cycle of obsessions and compulsions that significantly impact patients' quality of life. Suppressing obsessions and stopping compulsive behaviors requires executive control [2] and specifically inhibitory control [3], [4], [5]. Alertness is one factor that affects inhibitory control [6]. Hence, understanding how changes in alertness affect inhibitory control in individuals with OCD is crucial, as it sheds light on their ability to manage OCD symptoms.

Inhibitory control is an executive mechanism that underlies our ability to suppress behaviors, thoughts, and emotions [7], [8], [9] and to achieve and maintain goal-directed behavior. Several studies suggest that inhibitory deficits play a crucial role in the etiology of OCD [10], [11], [12]; other studies argue that these deficits might not be consistent in all patients [13], are small and clinically irrelevant [14], or are the result of the symptoms and not the cause [15]. Whether there is a fundamental deficit or not, the necessity of inhibitory control in managing unwanted behaviors, thoughts, or emotions and thereby mitigating OCD symptoms, is widely acknowledged and well-documented in both behavioral [7], [16], [17], [18] and neuroimaging studies [19].

One significant factor that can affect inhibitory control is alertness [20], which is a bottom-up attentional system, mediated by norepinephrine released from the locus coeruleus [21], possibly via increasing sensitivity to external cues [22]. The locus coeruleus has a direct link with the right inferior frontal gyrus, a brain region that mediates inhibitory control [23], [24], and inhibitory control improves with increased neuroepinephrine [25]. Behavioral studies in healthy adults have also shown that increasing alertness can improve inhibition [6]. In OCD patients, alertness has been associated with the disorder’s phenotype [26], [27]. Specifically, through its effect on inhibitory control, alertness has been shown to enhance the ability to resist symptoms [28], [29]. For example, Naftalovich et al. [30] manipulated alertness levels using caffeine and observed its positive impact on the ability to resist compulsive urges in OCD patients (see also [26]). Similarly, preliminary evidence from studies on light therapy, which affects alertness by regulating circadian rhythms, has shown promising outcomes in alleviating OCD severity [31].

While alertness levels are influenced by various factors, circadian rhythm has a major effect on fluctuations in alertness levels throughout the day. It constitutes a daily cycle involving major physiological processes including wakefulness and cognitive abilities [32]. Individuals vary in their circadian rhythm patterns, which are driven by the biological clocks and reflected in their *chronotype* — individuals’ optimal time of day for different activities [33], [34], [35], [36]. While chronotype has been defined in various ways, it is most understood as the temporal organization of daily activities, encompassing both actual behavior (influenced by external factors) and personal preferences (when such factors are absent [37], [38]. Chronotype is a continuum [39] ranging from strong morningness tendencies (‘morning types’) to strong eveningness tendencies (‘evening types’) based on the peak alertness time during the day. Substantial evidence indicate that chronotype impact various physiological and psychological processes [39], [40].

Although research on the relationship between chronotype and OCD is still emerging, a few studies have demonstrated links between circadian rhythms, sleep disturbances, and OCD in both clinical [41] and non-clinical samples [42], [43], [44], [45]. More specifically, regarding the role of chronotype in OCD, the seminal work of Cox and colleagues in a large non-clinical sample revealed an association between OC symptoms (measured by the OCI-R) and eveningness chronotype, though symptoms of depression (measured by the DASS-21) explained this relationship to a greater extent [43]. Notably, eveningness chronotype predicted an increase in OC symptoms two months later, an effect partially mediated by worsening sleep disturbances (see also [46] for evidence for connection between eveningness and disrupted sleep and negative affect in a clinical sample of OCD). In a more recent study, Cox et al. [47] found eveningness chronotype was associated with worst anxiety symptoms in the evening among non-clinical adults with self-reported anxiety or OC symptoms. In the first longitudinal study of patients with OCD, Naftalovich et al. [48] monitored OCD patients for 7 days and showed that symptoms were more frequent and more severe in patients’ non-optimal time — morning for evening-type patients and evening for morning-type patients. Although this effect was hypothesized to be related to inhibitory control levels, inhibitory control was not tested.

Taken together, the literature supports the hypothesis that alertness levels, influenced by one's chronotype and time of day, affect the ability to inhibit OCD symptoms. In other words, individuals with OCD may find it easier to inhibit symptoms during their optimal time of day than during their non-optimal time. This hypothesis has received support from a recent pilot study [49] in OCD patients that utilized a novel Symptom-Provocation Stop-Signal Task (SP-SST), which assesses inhibitory control in response to neutral images and to individually tailored obsession-inducing images, aimed to provoke OCD symptoms. This study found that chronotype and time of day affected inhibitory control on this task, but the sample size was small (10 morning type and 15 evening type) and chronotype was determined in a non-standard way (i.e., using the median score of the Morningness-Eveningness Questionnaire (MEQ [35]). As a result, individuals with intermediate tendencies toward morning or evening chronotype were included and analyses testing the continuous association between chronotype and inhibitory control were precluded.

The goal of the current study was to further test the hypothesis that alertness levels, influenced by one’s chronotype and time of day, affect inhibitory control by using standard methods for assessing chronotype and a larger sample. To that aim, treatment-seeking OCD patients completed SP-SST sessions every morning and every evening for four consecutive days. This four-day duration was chosen to balance feasibility with the need for adequate data collection to ensure reliability while minimizing attrition and fatigue. We hypothesized: (1) that OCD patients will exhibit worse inhibitory control in response to symptom-provoking images compared to neutral ones; and (2) that inhibitory control will be better at the ideal time (morning for morning type, evening for evening type) compared to the non-ideal time, and that this effect will be greater in the symptom-provocation condition compared with the neutral condition.

## 1 Methods

### 1.1 Study design

The study was conducted as part of a larger treatment study (no data on that sample was yet published) and approved by the institutional review board (XXXX-23012023). After providing written informed consent, participants completed baseline clinical assessments and self-report questionnaires. Subsequently, the SP-SST was installed on patients' personal computers, and they completed the task from home for four days.

### 1.2 Participants

Ninety-three adults between the ages of 18 and 67 were recruited for a treatment study through advertisements, word of mouth, and clinical referrals. To be eligible, individuals had to have a principal diagnosis of OCD and a Yale-Brown Obsessive Compulsive Scale (Y-BOCS [50]) score of ≥ 16. Table 1 describes demographic and clinical characteristics of the sample. Exclusion criteria included current or past manic episodes, psychosis, prominent suicidal ideation, substance abuse or dependence in the past 12 months, severe current major depressive disorder, or any neurological disorder. Patients were not excluded if they had comorbid anxiety disorder or mild-to-moderate depression, as long as OCD symptoms were the most severe and impairing of the pertinent diagnoses. Patients on psychiatric medication were eligible if they had maintained a stable dose for at least six weeks prior to and throughout the study. Eligibility was determined by trained clinical psychologists as described below. A power analysis using G*Power [51] indicated that a sample of 93 OCD patients would be sufficient to detect within- factors interaction with power > 95 % to test a small-medium effect size (based on our pilot study [49]) with a Type I error α < .05.

**Table 1:** Demographics and clinical characteristics.

	Morning type (N = 17)	Intermediate type (N = 39)	Evening type (N = 35)	Morning vs. Evening
Session completed (mean, SE)	6.53 (0.24)	6.73 (0.17)	6.57 (0.20)	*t*(50) = 0.13, *p = .90*
**Demographics**				
Age (years; mean, SE)	29.82 (1.92)	31.02 (1.69)	29.43 (1.68)	*t*(50) = 0.14, *p = .89*
Education (years; mean, SE)	13.41 (0.47)	14.37 (0.31)	13.50 (0.26)	*t*(49) = 0.18, *p = .86*
Gender (n female/male)	9/8	18/23	19/16	*χ*^2^< 0.01, *p* = .93
Medication (n, % on SRI/NSRI)	4 (23.50 %)	18 (43.90 %)	18 (51.40 %)	*χ*^2^< 3.15, *p* = .08
**Clinical Characteristics**				
MEQ (mean, SE)	66 (1.27)	49.10 (0.69)	32.77 (1.10)	*t*(50) = 18.29, p < .01 *
Y-BOCS (mean, SE)	27.47 (0.97)	28.49 (0.76)	29.17 (0.81)	*t*(50) = 1.26, *p = .21*
OCI-R (mean, SE)	29.47 (1.95)	31.54 (1.91)	33.77 (1.78)	*t*(50) = 1.49, *p = .14*
BDI-II (mean, SE)	17.76 (2.09)	17.59 (1.49)	21.29 (1.77)	*t*(50) = 1.20, *p = .24*

SRI = serotonin reuptake inhibitors; Y-BOCS = Yale-Brown Obsessive Compulsive Scale; MEQ = morningness-eveningness Questionnaire; OCI-R = obsessive-compulsive inventory – revised; BDI-II = Beck Depression inventory – II); SE = 1 standard error of the mean. Comparisons between morning and evening types are described in the Statistical Analyses section. Note: one participant refused to disclose years of education.

### 1.3 Clinical assessments

Diagnoses and clinical assessments were conducted by trained independent clinical psychologists with expertise in OCD who had no other contact with study patients. Independent evaluators conducted weekly reliability training. After a brief phone screening (conducted by a research assistant), potential participants were assessed to confirm eligibility using the Structured Clinical Interview for DSM-V (SCID-5 [52]) and the Y-BOCS [50]. Next, patients completed the Obsessive–Compulsive Inventory—Revised (OCI-R [53]), a self-report measure of OCD symptom severity, the Beck Depression Inventory—II (BDI-II [54]) to assesses the severity of depressive symptoms, and the MEQ [35] to determine their chronotype. The MEQ measures individual's circadian preference, reflecting the peak alertness patterns based on one’s chronotype. The scores range from 16 (definitely evening) to 86 (definitely morning), with scores higher than 58 representing “morning types”, scores lower than 42 representing “evening types”, and scores between 42 and 58 representing an “intermediate type”. Internal consistency for this questionnaire is between a Cronbach’s alpha of.77–.80 [55].

### 1.4 The symptom-provocation stop-signal task (SP-SST)

**Fig. 1:**
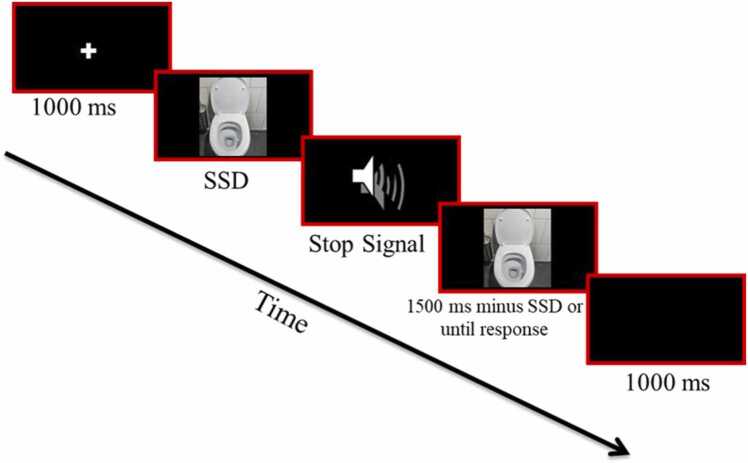
The symptom-provocation stop-signal task (SP-SST). Note An example of a symptom-provocation stop-signal trial.

The classic stop-signal task is a validated tool to measure inhibitory control [8], [56] and online versions of the task have shown similar high reliability [56], [57]. The SP-SST (Fig. 1) was modified from the classic stop-signal task using OpenSesame 3.0 (using Python 2.7.6), to measure inhibition related to OCD symptoms. Each session included 400 trials, each started with a 1000 ms fixation, followed by a target picture presented for 1500 ms or until a key press. Patients were asked to respond to the background color of the picture (discriminate between light and dark) by pressing the ‘z’ or ‘m’ on the computer keyboard. In 50 % of the trials, personalized OCD-provoking pictures were presented (e.g., a picture of a skin infection for a patient with skin-related contamination intrusions), whereas the other 50 % were neutral trials (e.g., flowers, houses). In 25 % of the trials, a stop-signal (an auditory tone, 750 Hz, 75 ms) appeared 200 ms after the presentation of the target picture (stop-signal delay; SSD), signaling that the response should be inhibited. The instruction indicated to respond with the corresponding index finger, as quickly and accurately as possible and not to wait for a potential stop-signal. The trial order was random. Each trial ended with a 1000 ms inter-trials interval. To personalize the task, the symptom-provoking pictures were selected by the evaluator and patient together at the end of the clinical assessment. Stimuli were chosen to effectively trigger the urge to do a compulsion without causing overwhelming anxiety [58]. Images were obtained through different resources, including the International Affective Picture System (IAPS [59]), online search engines (e.g., Google image search), and different open text-to-image artificial intelligence tools.

For four consecutive days, participants were instructed to complete three task sessions daily, approximately in the morning, at noon, and in the evening. Participants were instructed to complete the task in a quiet, private room, free from distractions such as phones, with clear and detailed guidance provided to ensure understanding and compliance. For the purposes of this study, only the morning and evening sessions were included in the analyses, and only as long as they were completed between 5 and 12 pm (morning) or between 5pm and 1am (evening). These time slots typically align with morning and evening peak alertness and cognitive function [60].

Stop-signal reaction time (SSRT), which is a reliable and valid measure for inhibitory control, was calculated using the integration method [56] for each condition (natural vs. symptom-provocation) separately. No-stop-signal trial RTs were determined by the *n*^th^ RT, that is, N (number of correct no-stop-signal trials) × p(response|signal), which was calculated for each participant in each condition separately. SSRT was then calculated as the *n*^th^ RT - SSD.

#### 1.4.1 Statistical analyses

To assess differences in demographic and clinical variables (Table 1), chi-square tests were used for categorical variables (gender and SRI status) and independent *t*-tests were used for continuous variables (session completed, age, education, Y-BOCS, MEQ, OCI-R, and BDI-II).

SSRT was calculated for each participant at each condition. Participants with extreme SSRTs (>3 SDs from the condition’s mean) were excluded from further analyses: this included one participant from the morning neutral condition; two from the symptom-provocation morning condition, one from the neutral evening condition, and one from all four conditions (and thus from all analyses).

To investigate our main a-priori hypotheses, we carried out four repeated measure correlation analyses between MEQ scores and SSRT within each valence condition (symptom-provocation vs. neutral) and each time of day (morning vs. evening). Finally, to investigate our a-priori hypothesis using categorial chronotype, SSRT data was subjected to a three-way mixed model analysis of variance (ANOVA) with valence condition and time of day as within-subject factors and chronotype group (morning type vs. evening type) as a between-subject factor. The significant interaction was further analyzed using interaction contrasts to test our a-priori hypotheses. An alpha level of 0.05 was considered the threshold for statistical significance, and all tests were two-tailed. All data necessary to reproduce the reported results is publicly available (*Mendeley Data, V1*, doi: 10.17632/r5fgbb4bjd.1).

## 2 Results

All participants completed at least five out of eight SP-SST sessions, with 82 % completing 6 or more sessions.^1^ As can be seen in Table 1, there were no significant differences between the groups in any demographic or clinical variables other than MEQ. There was a higher number of individuals with an evening chronotype compared to morning chronotype.

**Fig. 2:**
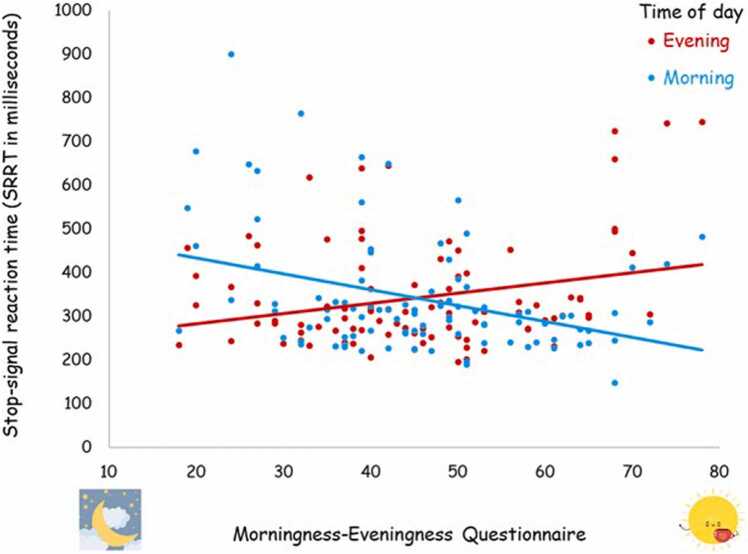
Correlations between the chronotype and inhibitory control at the morning and evening time-points for the symptom-provocation condition. Note. Lower scores on the MEQ represent a tendency toward evening chronotype and higher scores represent a tendency toward morning chronotype. Longer SSRT reflects less efficient inhibitory control.

For our main hypothesis, using MEQ as a continuous measure, there was no significant correlation for the neutral condition between chronotype and SSRT (morning time-point, *r*(91)*=* -.02, *p* = .83; evening time-point, *r*(91)*=* .15, *p* = .17). However, in the symptom-provocation condition, there was a significant negative correlation in the morning time-point, *r*(90)*=* -.30, *p* < .01, and a significant positive correlation in the evening time-point, *r*(92)*=* .26, *p* = .01 (Fig. 2).

**Fig. 3:**
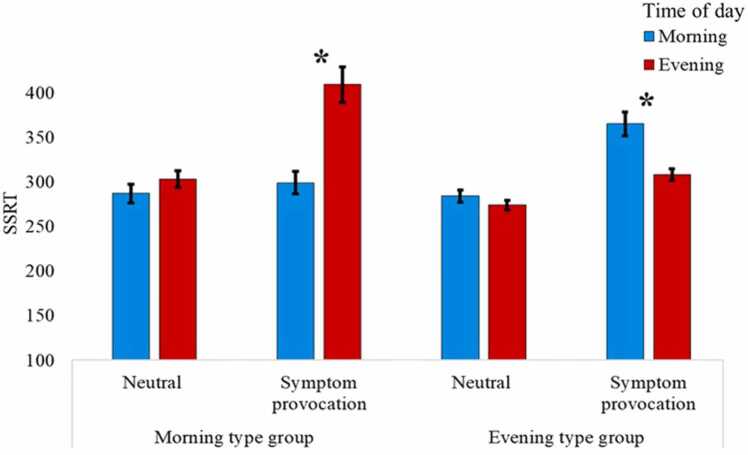
*Results by chronotype group. Note.* Upper panel – Method. An example of a trial of the symptom-provocation stop-signal task (SP-SST). Lower panel – Results. Mean stop-signal reaction time (SSRT) as a function of time of day and chronotype group, for the neutral and symptom-provocation conditions. Time of day represents measurement time. Error bars represent one standard error from the mean.

For our secondary analysis, the ANOVA yielded a main effect for valence, *F*_(1,46)_= 31.88, *p* < .001, $\eta_{p}^{2}$= .41, indicating longer SSRT to the symptom-provocation condition compared with the neutral condition. However, there were no main effects for time of day, *F*_(1,46)_= 2.76, *p* = .11, $\eta_{p}^{2}$= .06, nor for chronotype group, *F*_(1,46)_= 0.19, *p* = .89, $\eta_{p}^{2}$< .001. The three-way interaction between valence condition, time of day, and chronotype group was significant, *F*_(1,46)_= 9.98, *p* < .01, $\eta_{p}^{2}$= .18 (Table 2; Fig. 3). To examine this interaction, repeated measures two-way ANOVAs were conducted on SSRT data for each chronotype group separately, with valence and time of day as within-subject factors.

**Table 2:** Results.

		Morning type group		Intermediate group		Evening type group	
Session		Morning	Evening	Morning	Evening	Morning	Evening
nsRT	Neutral	587 (33)	580 (32)	559 (20)	536 (18)	549 (19)	540 (20)
	Symptom-provocation	594 (28)	651 (45)	614 (23)	588 (19)	637 (29)	616 (27)
nsACC	Neutral	.98 (.01)	.99 (.01)	.99 (.01)	.99 (.01)	.99 (.01)	.99 (.01)
	Symptom-provocation	.97 (.01)	.98 (.01)	.99 (.01)	.99 (.01)	.99 (.01)	.99 (.01)
SSRT	Neutral	287 (21)	303 (19)	265 (12)	270 (10)	284 (14)	274 (11)
	Symptom-provocation	299 (25)	409 (40)	318 (15)	313 (14)	365 (27)	308 (13)
p(r|s)	Neutral	.35 (.07)	.45 (.06)	.33 (.04)	.37 (.03)	.34 (.04)	.37 (.03)
	Symptom-provocation	.29 (.04)	.32 (.05)	.26 (.02)	.27 (.02)	.28 (.03)	.30 (.03)

*Note.* Mean reaction time in milliseconds (one standard error of the mean); nsRT = reaction time of correct responses for no-stop trials; nsACC = accuracy rate for no stop-signal trials; SSRT = stop-signal reaction time; p(r|s) = proportion of erroneous responses to stop-signal trials.

In the morning chronotype group, we found a main effect for valence condition, *F*_(1,14)_= 4.80, *p* = .04, $\eta_{p}^{2}$= .26, indicating longer SSRT in the symptom-provocation condition compared with the neutral condition, and a main effect for time of day, *F*_(1,14)_= 9.01, *p* = .01, $\eta_{p}^{2}$= .39, indicating longer SSRTs in the evening compared to the morning. The two-way interaction between valence condition and time of day was marginally significant, *F*_(1,14)_= 3.48, *p* = .08, $\eta_{p}^{2}$= .20. Planned comparisons for this interaction, using paired-samples *t*-tests revealed longer SSRT in the evening compared to the morning in the symptom-provocation condition, *t*_(15)_= 3.57, *p* < .01, Cohen’s *d* = .0.89,but no modulation of SSRT by time of day in the neutral valence condition, *t*_(15)_= .67, *p* = .51, Cohen’s *d* = 0.17.

In the evening chronotype group, we found a main effect for valence condition, *F*_(1,32)_= 52.20, *p* < .001, $\eta_{p}^{2}$= .62, indicating longer SSRT to the symptom-provocation condition compared with the neutral condition, and no main effect for time of day, *F*_(1,32)_= 2.41, *p* = .13, $\eta_{p}^{2}$= .07. The two-way interaction between valence condition and time of day was significant, *F*_(1,32)_= 5.62, *p* = .02, $\eta_{p}^{2}$= .15. Planned comparisons for this interaction, using paired-samples *t*-tests revealed shorter SSRT in the evening compared to the morning in the symptom-provocation condition, *t*_(33)_= 2.18, *p* = .03, Cohen’s *d* = .0.37, but no modulation of SSRT by time of day in the neutral valence condition, *t*_(33)_= 0.74, *p* = .47, Cohen’s *d*= 0.13 (Table 2 & Fig. 3).^2^

## 3 Discussion

The current study assessed inhibitory control to neutral vs. symptom-provoking images in a large sample of treatment-seeking OCD patients as a function of chronotype and time of day. As hypothesized, we observed reduced inhibitory control (as measured by longer SSRTs) in symptom-provocation trials compared to neutral trials. In addition, we also found that inhibitory control to symptom-provocation trials was significantly affected by the interaction between chronotype and time of day: there was a significant correlation between inhibitory control in the symptom provocation condition and chronotype, in both the morning and evening time-points. In addition, a categorical analysis revealed that patients with morning-type chronotype had better inhibition in the morning compared to the evening and vice versa. Together, these findings demonstrate that behavioral inhibition to symptom-provocation cues is influenced by the interaction between chronotype and time of day. Contrary to our hypotheses, in the neutral condition, inhibitory control was not affected by chronotype and time of day.

Our finding that symptom-provocation reduces inhibitory control is consistent with prior literature [61]. Different mechanistic ideas have been proposed for this effect. One proposed mechanism is that symptom-provocation cues trigger negative emotions, which have been shown in healthy adults and anxiety patients to have a detrimental effect on behavioral inhibition [9], [18], [62]. For example, Adams [63] found that disorder-relevant cues led to greater difficulty in inhibiting actions in participants with high and low contamination fears. Functional imaging studies in OCD patients further support this relationship, revealing increased amygdala activation and enhanced connectivity between the amygdala and the prefrontal cortex (which underlies less effective inhibitory control) during symptom-provocation [64], [65]. Indeed, a recent model of OCD suggested that anxiety and other negative emotions (which are provoked by the OCD-related cues) might lead to reduced inhibitory control, which in turn decreases the ability to resist compulsions, perpetuating the vicious cycle of the disorder [66]. A second possible mechanism suggests that symptom-provocation may lead to reduced inhibitory control because obsessions overload OCD patients’ cognitive system [15], [67], [68]. Finally, the detrimental effect of symptom-provocation on inhibitory control may be understood in terms of increased action tendencies. It has been shown that emotions can elicit pre-potent responses or action tendencies [69]. For example, glee is associated with approach behaviors and distress is associated with moving away from a stimulus [70]. Thus, by eliciting emotions, OCD symptom-provocation might induce potent action tendencies that are challenging to inhibit [71], [72]. Taken together, these models suggest that while inhibition is needed to reduce OCD symptoms, OCD symptoms might also lead to reduced inhibition [66]. Hence, the detrimental effect of symptom-provocation on inhibitory control in OCD might potentially fuel the cycle of obsessions, distress, and compulsions.

Our finding that chronotype affects inhibitory control to symptom-provocation cues as a function of time of day, confirms and extends our pilot work [49], lending additional support for the notion that OCD patients’ ability to resist an urge to perform a compulsion can is influenced by their current alertness levels, as reflected by their chronotype and time of day. While this finding is not consistent with Cox et al. [47], who reported that in a non-clinical sample, individuals with an evening chronotype and high OC symptoms experienced greater anxiety in the evening, it does correspond with findings of Naftalovich et al. [48] who found, in a sample of treatment-seeking OCD patients, that OCD symptoms were most severe in the morning for evening-type patients and vice versa. Our results also align with research indicating that some individuals with depression and an eveningness chronotype tend to exhibit worst symptoms in the morning [73].

That daily fluctuations in the ability to execute inhibition to OCD symptom can be predicted by chronotype and time of day might have important clinical implications. First, clinicians can explain to patients how knowing their chronotype can help them anticipate times of the day when resisting rituals may be more challenging. Second, clinicians can consider chronotype-alignment during exposure and response prevention treatment for optimal benefit in scheduling treatment sessions and homework. For example, if the patient has a morning chronotype, the clinician may start by assigning more intense exposures during the morning hours, when patient’s inhibitory control is at its best, and then move to exposures during the evening, when patient’s inhibitory control will be less efficient.

There were two unexpected findings. First, unlike our pilot study [49] the interaction between chronotype and time of day did not affect inhibitory control in the neutral condition. This finding contradicts some initial evidence from non-clinical samples showing better performance in inhibitory tasks in the ideal vs. non-ideal time [74], although some studies found this effect only in brain measures, with no behavioral manifestation [60]. One potential explanation for our finding is a ceiling effect in the neutral condition. The relatively short SSRT and low rate of erroneous responses to stop signals (p(r|s)), seen in the neutral condition, support this notion. In the symptom-provocation condition, the cues caused an additional interference, preventing a similar ceiling effect from occurring. Further investigation into the effect of chronotype and time of day on inhibitory control in a neutral stop-signal task is needed. The second surprising finding was the low proportion of OCD individuals with morning chronotype despite the fact that there is a significantly higher proportion of morning chronotype in the general population [34]. This finding contrasts with one study that found no increased prevalence of evening chronotypes among non-clinical individuals with high self-reported OC symptoms [47]. However, it aligns with previous research linking OCD symptom severity to later bedtimes [75] and two other studies reporting a higher prevalence of evening chronotypes in OCD patients [43], [76]. Notably, one of these latter studies [43] suggested that the relationship between OCD and eveningness chronotype is better explained by levels of depression. The notion that individuals with OCD might exhibit significantly more evening-type chronotype compared to the general population should be further investigated.

The current study benefits from a well-diagnosed, well-characterized, and well-diagnosed sample of treatment-seeking adults with OCD, the implementation of a novel task incorporating validated measures of inhibitory control with individually tailored symptom-provocation cues, and the use of standardized MEQ cutoffs to classify morning and evening chronotype. Limitations include a relatively small 'morning-type' group (which may be inherent to the disorder), a mixed sample in terms of age range and medication use (although all participants were stable for at least six weeks), the absence of a standardized (or individualized) time of day for task completion, and the at-home completion of testing sessions, which may have introduced variability despite detailed instructions provided to improve compliance. Future studies should also collect general and specific (night before) sleep measures to determine their influence, if any, on these findings.

In conclusion, the current study investigated the role of chronotype and time of day on inhibitory control in adults with OCD. We found that individuals with OCD exhibit reduced inhibitory control when confronted with symptom-provoking stimuli compared to neutral stimuli, potentially revealing the vicious cycle of OCD, wherein symptom-provoking cues diminish inhibitory control and consequently reduce patients' ability to resist compulsions. We also found that inhibitory control in these individuals with OCD when confronted with these stimuli was affected by chronotype and time of day. This suggests that heightened alertness—by increasing the chances of successful inhibition--may constitute a new way to mitigate the vicious cycle of OCD.

## Ethical standards

The authors assert that all procedures contributing to this work comply with the ethical standards of the relevant national and institutional committees on human experimentation and with the Helsinki Declaration of 1975, as revised in 2008. The study was approved by the institutional review board (HUJI-2301-2023).

## Declaration of Competing Interest

The authors declare the following financial interests/personal relationships which may be considered as potential competing interests: Eyal Kalanthroff reports financial support was provided by Israel Science Foundation (EK; grant no. 1341/18). In the last three years, Dr. Simpson has received a stipend from the American Medical Association to serve as Associate Editor of JAMA Psychiatry and royalties from Cambridge University Press and UpToDate, Inc. If there are other authors, they declare that they have no known competing financial interests or personal relationships that could have appeared to influence the work reported in this paper.

## Footnotes

1 A breakdown of the data by group revealed that neither group completed significantly more sessions at either time point.

2 For the intermediate group, a similar analysis yielded a main effect for valence condition, *F*_(1,39)_= 72.26, *p* < .001, $\eta_{p}^{2}$= .65, no main effect for time of day, *F*_(1,39)_= 0.01, *p* = .99, and no two-way interaction, *F*_(1,39)_= 2.16, *p* = .13.

## References

[1] American Psychiatric Association (2013).

[2] Fitzgerald K.D., Schroder H.S., Marsh R. (2021). Cognitive control in pediatric obsessive-compulsive and anxiety disorders: brain-behavioral targets for early intervention. Biol Psychiatry.

[3] Anholt G.E., Linkovski O., Kalanthroff E. (2012). If I do it, it must be important: integrating basic cognitive research findings with cognitive behavior theory of obsessive-compulsive disorder. Psicoter Cogn Comport.

[4] Gillan C.M., Kalanthroff E., Evans M., Weingarden H.M., Jacoby R.J., Gershkovich M. (2020). Comparison of the association between goal-directed planning and self-reported compulsivity vs obsessive-compulsive disorder diagnosis. JAMA Psychiatry.

[5] Linkovski O., Kalanthroff E., Henik A., Anholt G.E. (2013). Did I turn off the stove? Good inhibitory control can protect from influences of repeated checking. J Behav Ther Exp Psych.

[6] Weinbach N., Kalanthroff E., Avnit A., Henik A. (2015). Can arousal modulate response inhibition*?*. J Exp Psychol Learn Mem Cogn.

[7] Anderson M.C., Hulbert J.C. (2021). Active forgetting: adaptation of memory by prefrontal control. Annu Rev Psychol.

[8] Logan G.D., Cowan W.B. (1984). On the ability to inhibit thought and action: a theory of an act of control. Psychol Rev.

[9] Verbruggen F., De Houwer J. (2007). Do emotional stimuli interfere with response inhibition? Evidence from the stop signal paradigm. Cogn Emot.

[10] Chamberlain S.R., Blackwell A.D., Fineberg N.A., Robbins T.W., Sahakian B.J. (2005). The neuropsychology of obsessive-compulsive disorder: the importance of failures in cognitive and behavioural inhibition as candidate endophenotypic markers. Neurosci Biobehav Rev.

[11] Mar K., Townes P., Pechlivanoglou P., Arnold P., Schachar R. (2022). Obsessive compulsive disorder and response inhibition: meta-analysis of the stop-signal task. J Psychopathol Clin Sci.

[12] Morein-Zamir S., Fineberg N.A., Robbins T.W., Sahakian B.J. (2010). Inhibition of thoughts and actions in obsessive-compulsive disorder: extending the endophenotype?. Psychol Med.

[13] Kalanthroff E., Teichert T., Wheaton M.G., Kimeldorf M.B., Linkovski O., Ahmari S.E. (2017). The role of response inhibition in medicated and unmedicated obsessive-compulsive disorder patients: evidence from the stop-signal task. Depress Anxiety.

[14] Abramovitch A., Abramowitz J.S., Mittelman A. (2013). The neuropsychology of adult obsessive–compulsive disorder: A meta-analysis. Clin Psychol Rev.

[15] Abramovitch A., Cooperman A. (2015). The cognitive neuropsychology of obsessive-compulsive disorder: a critical review. J Obsessive Compuls Relat Disord.

[16] Akerman A., Naftalovich H., Akiva-Kabiri L., Kalanthroff E. (2020). Inhibiting the emergence of involuntary musical imagery: Implications for improving our understanding of intrusive thoughts. Cogn Ther Res.

[17] Bartholomew M.E., Heller W., Miller G.A. (2021). Inhibitory control of emotional processing: theoretical and empirical considerations. Int J Psychophysiol.

[18] Kalanthroff E., Cohen N., Henik A. (2013). Stop feeling: Inhibition of emotional interference following stop-signal trials. Front Hum Neurosci.

[19] Viard A., Flament M.F., Artiges E., Dehaene S., Naccache L., Cohen D. (2005). Cognitive control in childhood-onset obsessive–compulsive disorder: a functional MRI study. Psychol Med.

[20] Petersen S.E., Posner M.I. (2012). The attention system of the human brain: 20 years after. Annu Rev Neurosci.

[21] Posner M.I., Rothbart M.K. (1992). In: Milner AD, Rugg MD, editors. The neuropsychology of consciousness.

[22] Hayat H., Regev N., Matosevich N., Sales A.C., Paredes-Rodriguez E., Krom A.J. (2020). Locus coeruleus norepinephrine activity mediates sensory-evoked awakenings from sleep. Sci Adv.

[23] Aston-Jones G., Cohen J.D. (2005). An integrative theory of locus coeruleus-norepinephrine function: adaptive gain and optimal performance. Annu Rev Neurosci.

[24] Aston-Jones G., Waterhouse B. (2016). Locus coeruleus: from global projection system to adaptive regulation of behavior. Brain Res.

[25] Zhukovsky P., Morein-Zamir S., Ziauddeen H., Fernandez-Egea E., Meng C., Regenthal R. (2022). Prefrontal cortex activation and stopping performance underlie the beneficial effects of atomoxetine on response inhibition in healthy volunteers and those with cocaine use disorder. Biol Psychiatry Cogn Neurosci Neuroimag.

[26] Koran L.M., Aboujaoude E., Gamel N.N. (2009). Double-blind study of dextroamphetamine versus caffeine augmentation for treatment-resistant obsessive-compulsive disorder. J Clin Psychiatry.

[27] Nota J.A., Gibb B.E., Coles M.E. (2014). Obsessions and time of day: a self-monitoring study in individuals with obsessive-compulsive disorder. J Cogn Psychother.

[28] Kalanthroff E., Linkovski O., Weinbach N., Pascucci O., Anholt G.E., Simpson H.B. (2017). What underlies the effect of sleep disruption? The role of alertness in obsessive-compulsive disorder (OCD). J Behav Ther Exp Psychiatry.

[29] Nota J.A., Schubert J.R., Coles M.E. (2016). Sleep disruption is related to poor response inhibition in individuals with obsessive–compulsive and repetitive negative thought symptoms. J Behav Ther Exp Psychiatry.

[30] Naftalovich H., Tauber N., Kalanthroff E. (2020). But first, coffee: the roles of arousal and inhibition in the resistance of compulsive cleansing in individuals with high contamination fears. J Anxiety Disord.

[31] Coles M.E., Stewart E. (2019). Circadian zeitgebers and treatment outcome in inpatient programs for obsessive-compulsive disorder (OCD): a pilot study. Chrono Int.

[32] Tonetti L., Natale V., Randler C. (2015). Association between circadian preference and academic achievement: A systematic review and meta-analysis. Chrono Int.

[33] Dijk D.J., Duffy J.F., Czeisler C.A. (1992). Circadian and sleep/wake dependent aspects of subjective alertness and cognitive performance. J Sleep Res.

[34] Fischer D., Lombardi D.A., Marucci-Wellman H., Roenneberg T. (2017). Chronotypes in the US–influence of age and sex. PLoS One.

[35] Horne J.A., Ostberg O. (1976). A self-assessment questionnaire to determine morningness-eveningness in human circadian rhythms. Int J Chrono--.

[36] Matchock R.L., Mordkoff T.J. (2009). Chronotype and time-of-day influences on the alerting, orienting, and executive components of attention. Exp Brain Res.

[37] Zou H., Zhou H., Yan R., Yao Z., Lu Q. (2022). Chronotype, circadian rhythm, and psychiatric disorders: Recent evidence and potential mechanisms. Front Neurosci.

[38] Montaruli A., Castelli L., Mulè A. (2021). Biological rhythm and chronotype: new perspectives in health. Biomolecules.

[39] Natale V., Cicogna P. (2002). Morningness-eveningness dimension: is it really a continuum?. Pers Individ Dif.

[40] Carasso S., Fishman B., Lask L.S., Shochat T., Geva-Zatorsky N., Tauber E. (2021). Metagenomic analysis reveals the signature of gut microbiota associated with human chronotypes. bioRxiv.

[41] Cox R.C., Olatunji B.O. (2022). Delayed circadian rhythms and insomnia symptoms in obsessive-compulsive disorder. J Affect Disord.

[42] Cox R.C., Olatunji B.O. (2016). Sleep disturbance and obsessive-compulsive symptoms: results from the national comorbidity survey replication. J Psychiatr Res.

[43] Cox R.C., Tuck B., Olatunji B.O. (2018). The role of eveningness in obsessive-compulsive symptoms: cross-sectional and prospective approaches. J Affect Disord.

[44] Schubert J.R., Coles M.E. (2013). Obsessive-compulsive symptoms and characteristics in individuals with delayed sleep phase disorder. J Nerv Ment Dis.

[45] Alvaro P.K., Roberts R.M., Harris J.K., Bruni O. (2017). The direction of the relationship between symptoms of insomnia and psychiatric disorders in adolescents. J Affect Disord.

[46] Simor P., Harsányi A., Csigó K., Miklós G., Lázár A.S., Demeter G. (2018). Eveningness is associated with poor sleep quality and negative affect in obsessive–compulsive disorder. J Behav Addict.

[47] Cox R.C., Wright K.P., Jr, Axelsson J., Balter L.J. (2024). Diurnal variation in anxiety and activity is influenced by chronotype and probable anxiety-related disorder status. Psychiatry Res.

[48] Naftalovich H., Anholt G.E., Keren R. (2021). Waxing and waning: the roles of chronotype and time of day in predicting symptom fluctuations in obsessive-compulsive disorder using a daily-monitoring design. J Psychiatr Res.

[49] Linkovski O., Naftalovich H., David M., Seror Y., Kalanthroff E. (2023). The effect of symptom-provocation on inhibitory control in obsessive-compulsive disorder patients is contingent upon chronotype and time of day. J Clin Med.

[50] Goodman W.K., Price L.H., Rasmussen S.A. (1989). The Yale-Brown obsessive-compulsive scale: I. Development, use, and reliability. Arch Gen Psychiatry.

[51] Faul F., Erdfelder E., Lang A.G., Buchner A.G. (2007). Power 3: a flexible statistical power analysis program for the social, behavioral, and biomedical sciences. Behav Res Methods.

[52] First M.B., Williams J.B.W., Karg R.S., Spitzer R.L. (2015). Structured clinical interview for DSM-5—Research version (SCID-5 for DSM-5, research version; SCID-5-RV). Am Psychiatr Assoc.

[53] Foa E.B., Huppert J.D., Leiberg S., Langner R., Kichic R., Hajcak G. (2002). The obsessive-compulsive inventory: development and validation of a short version. Psychol Assess.

[54] Beck A.T., Steer R.A., Brown G. (1996). Beck depression inventory–II. Psych Assess.

[55] Thun E., Bjorvatn B., Osland T., Steen V.M., Sivertsen B., Johansen T. (2012). An actigraphic validation study of seven morningness-eveningness inventories. Eur Psychol.

[56] Verbruggen F., Aron A.R., Band G.P., Beste C., Bissett P.G., Brockett A.T. (2019). A consensus guide to capturing the ability to inhibit actions and impulsive behaviors in the stop-signal task. eLife.

[57] Poulton A., Chen L.P., Dali G., Fox M., Hester R. (2022). Web-based independent versus laboratory-based stop-signal task performance: within-subjects counterbalanced comparison study. J Med Internet Res.

[58] Broekhuizen A., Vriend C., Wolf N., Koenen E.H., van Oppen P., van Balkom A.J., Visser H.A., van den Heuvel O.A. (2023). Poor insight in obsessive-compulsive disorder as a multifaceted phenomenon: evidence from brain activation during symptom provocation. Biol. Psychiatry: Cogn. Neurosci. Neuroimaging.

[59] Lang P., Bradley M.M. (2007). Handbook of Emotion Elicitation and Assessment.

[60] Song J., Feng P., Zhao X., Xu W., Xiao L., Zhou J. (2018). Chronotype regulates the neural basis of response inhibition during the daytime. Chrono-- Int.

[61] Kalanthroff E., Steinman S.A., Schmidt A.B., Campeas R., Simpson H.B. (2018). Piloting a personalized computerized inhibitory training program for individuals with obsessive-compulsive disorder. Psychotherapy and Psychosomatics.

[62] Keha E., Naftalovich H., Shahaf A., Kalanthroff E. (2024). Control your emotions: evidence for a shared mechanism of cognitive and emotional control. Cogn Emot.

[63] Adams T.G. (2015). Exposure to emotionally arousing, contamination-relevant pictorial stimuli interferes with response inhibition: Implication for obsessive–compulsive disorder. J Obsessive Compuls Relat Disord.

[64] Paul S., Beucke J.C., Kaufmann C., Mersov A., Heinzel S., Kathmann N. (2019). Amygdala–prefrontal connectivity during appraisal of symptom-related stimuli in obsessive–compulsive disorder. Psychol Med.

[65] Simon D., Adler N., Kaufmann C., Kathmann N. (2014). Amygdala hyperactivation during symptom provocation in obsessive–compulsive disorder and its modulation by distraction. NeuroImage Clin.

[66] Kalanthroff E., Wheaton M.G. (2022). An integrative model for understanding obsessive-compulsive disorder: merging cognitive behavioral theory with insights from clinical neuroscience. J Clin Med.

[67] Abramovitch A., Dar R., Hermesh H., Schweiger A. (2012). Comparative neuropsychology of adult obsessive-compulsive disorder and attention deficit/hyperactivity disorder: implications for a novel executive overload model of OCD. J Neuropsychol.

[68] Bednarek L., Glover S., Ma X., Pittenger C., Pushkarskaya H. (2024). Externally orienting cues improve cognitive control in OCD. J Behav Ther Exp Psychiatry.

[69] Frijda N.H. (1987). Emotion, cognitive structure, and action tendency. Cogn Emot.

[70] Rinck M., Becker E.S. (2007). Approach and avoidance in fear of spiders. J Behav Ther Exp Psychiatry.

[71] Dayan-Riva A., Berger A., Anholt G.E. (2019). Early cognitive processes in OCD: an ERP study. J Affect Disord.

[72] Linkovski O., Kalanthroff E., Henik A., Anholt G.E. (2016). Stop checking: repeated checking and its effects on response inhibition and doubt. J Behav Ther Exp Psychiatry.

[73] Chen Z., Zhao S., Tian S., Yan R., Wang H., Wang X. (2022). Diurnal mood variation symptoms in major depressive disorder associated with evening chronotype: evidence from a neuroimaging study. J Affect Disord.

[74] Lara T., Madrid J.A., Correa Á. (2014). The vigilance decrement in executive function is attenuated when individual chronotypes perform at their optimal time of day. PLoS One.

[75] Schubert J.R., Stewart E., Coles M.E. (2019). Later bedtimes predict prospective increases in symptom severity in individuals with obsessive-compulsive disorder (OCD): an initial study. Behav Sleep Med.

[76] Sakalli Kani A., Aksoy Poyraz C., Poyraz B.C., Bayar M.R., Akin E., Kose S. (2018). The role of affective temperaments and chronotype in pharmacotherapy response in patients with obsessive-compulsive disorder. Psychiatry Clin Psychopharmacol.

